# Duration of Protective Immunity in Sheep Vaccinated with a Combined Vaccine against Peste des Petits Ruminants and Sheep Pox

**DOI:** 10.3390/vaccines9080912

**Published:** 2021-08-16

**Authors:** Zhanat Amanova, Kuandyk Zhugunissov, Kainar Barakbayev, Zhanat Kondybaeva, Zhanna Sametova, Yeraly Shayakhmetov, Dastan Kaissenov, Kuanysh Dzhekebekov, Asankadyr Zhunushov, Yergaly Abduraimov, Kunsulu Zakarya, Yerbol Bulatov

**Affiliations:** 1 Research Institute Biological Safety Problems, Gvardeiskiy 080409, Kazakhstan; kuandyk_83@mail.ru (K.Z.); kainar7@mail.ru (K.B.); Zhanat.Kondybaeva@mail.ru (Z.K.); sametova_zh.zh@mail.ru (Z.S.); Eraly.Shax@gmail.com (Y.S.); k_dosik@mail.ru (D.K.); zhekebekov_87@mail.ru (K.D.); abduraimov_72@mail.ru (Y.A.); rkm_kz@mail.ru (K.Z.); erbol_km@mail.ru (Y.B.); 2 Institute of Biotechnology, National Academy of Sciences of the Kyrgyz Republic, Bishkek 720071, Kyrgyzstan; junushov@mail.ru

**Keywords:** combined vaccine, peste des petites ruminants, sheep pox, immunity

## Abstract

In this study, the ability of the combined vaccine against peste des petits ruminants (PPR) (Nigeria strain 75/1) and sheep pox (SPP) (NISKhI strain) to form a protective immune response for 12 months in Kazakh breed fine-fleeced sheep aged 6–12 months was demonstrated. The duration of the protective immunity of immunized sheep from PPR and from SPP was evaluated using a serum neutralization test (SNT), followed by testing of the resistance of vaccinated sheep to infection with the field strain Kentau-7 of the PPRV and the virulent strain A of the SPPV. The PPR antibody response was additionally measured by c-ELISA. A single immunization of sheep with a combined vaccine in a volume of 2.0 mL, containing the PPR and SPP vaccine viruses in the titers of 10^3.0^ TCID_50_/mL, provided reliable protection of animals from two infections simultaneously for 12 months (observation period). At the same time, in sheep immunized with the combined vaccine, antibodies of PPRV persisted for up to 12 months, with slight fluctuations. The combined vaccine induced 100% clinical protection against the field strain of PPRV and the virulent strain of SPPV in immunized sheep for up to 12 months, while unvaccinated animals became ill with the manifestation of clinical signs specific to PPRV and SPPV.

## 1. Introduction

A peste des petites ruminant (PPR) is a highly contagious, acute, re-emerging/developing disease in small ruminants. It is caused by a virus from the Paramyxoviridae family, Morbillivirus genus [1]. PPR is endemic throughout Africa, the Middle East, and large regions of Asia [2], and is currently reported in more than 70 countries in Africa, the Near and Middle East, as well as Central and East Asia, including Georgia in 2016 and Bulgaria in 2018 [3,4,5]. Due to its impact in April 2015, the Food and Agriculture Organization of the United Nations (FAO) and the Office International des Epizooties (OIE) launched a campaign to eradicate PPR globally by 2030 [2].

In the Republic of Kazakhstan (RK), the outbreak of PPR was officially recorded among small agricultural ruminants in the Turkestan region in 2003 [6]. The disease was reported in the country in 2005–2006 and 2014 [7,8]. Through the present introduction of PPR, it is highly possible in southeast Kazakhstan through the land boundary with China. PPR has become widespread in China since 2013, which is when the disease was allegedly introduced from the Republic of Tajikistan, according to the European Food Safety Authority (EFSA) and as shown by the results from the phylogenetic analysis [9].

Sheep pox (SPP) is a viral acute contagious disease caused by the virus from the Capripoxvirus genus, which belongs to the vast family of Poxviridae [10]. It is endemic in Central and Northern Africa, in the Near and Far East, as well as on the Indian subcontinent [11,12]. Over the past three decades, the epizootic situation in sheep and goat pox has been deteriorating in countries in Africa and Eurasia. During this period, the diseases were reported in over 70 countries across the globe [13]. The outbreak of SPP among small agricultural ruminants in 2019 in the western part of the Republic of Kazakhstan (RK) (Mangistau region) should be noted. Thanks to emergency measures undertaken by the veterinary services, the infection was localized [14]. Though the available monovalent PPR and SPP vaccines [15,16] are effective, the current PPR and SPP epizootic situation in RK requires improvements to be made to the existing prophylactic preparations against these infections. Thus, a new combined vaccine was developed in Kazakhstan to prevent both PPR and SPP simultaneously. The combined vaccines against PPR and SPP were developed earlier and were experimentally used with positive results in India [17], Cameroon [18], Morocco [19,20], Ethiopia [21], Egypt [22], and Russia [23]. Immunogenicity and the effectiveness of these vaccines are comparable to the same monovalent vaccines against PPR and SPP. Despite the formation of effective immunity, the available publications do not provide data on the assessment of the duration of post-vaccination immunity in animals vaccinated with the combined vaccines developed against these diseases. However, the vaccine strains used for the preparation of existing combined vaccines against PPR and SPP are sufficiently immunogenic and, being part of monovalent vaccines, create immunity in the once-immunized animals for at least 1 year.

Keeping in mind the long-term efficacy of the Nigeria 75/1 strain of the PPR virus (PPRV) and the NISKhI strain of the SPP virus (SPPV) in monovalent vaccines, a combined vaccine against PPR and SPP based on these strains was developed for the first time in the Republic of Kazakhstan.

As is known, the Nigeria 75/1 strain of the PPRV causes persistent immunity in once-immunized animals for up to 3 years, while the NISKhI strain of the SPPV can form protective immunity in the once-vaccinated animals for 1 year. The effectiveness of these strains, which are the main immunogens in the composition of the combined PPR and SPP vaccine developed by us, was a prerequisite for studying the duration of immunity in animals vaccinated with the new combined PPR and SPP vaccine for the Republic of Kazakhstan.

**Objective of this work was to assess the duration of immunity in sheep that were vaccinated with a combined vaccine against PPR and SPP developed in the Republic of Kazakhstan.**

## 2. Materials and Methods

### 2.1. Vaccine Virus

The combined vaccine against PPR and SPP was constructed, as previously described [24]. For the production of a combined vaccine against PPR and SPP, we used attenuated vaccine strains Nigeria 75/1 PPRV (GenBank: KY628761.1) and NISKhI SPPV (GenBank: AY077834.1), which were grown in the Vero cell line. This combination of strains was tested for the first time. During the production of the combined vaccine, suspensions of the Nigeria 75/1 PPRV and NISKhI SPPV vaccine strains with the same titer (at least 10^6.5^ lg TCID_50_/mL) were combined in an equal ratio of 1:1. A combination of peptone-sucrose stabilizer (in the final concentration of 3% peptone and 2% sucrose) was added to the prepared combined vaccine fluid of PPRV and SPPV in a ratio of 1:1. Then, the vaccine liquid was divided into aliquots in 2.0 mL ampoules and lyophilized for storage.

The combined vaccine was manufactured at the Research Institute for the Biological Safety Problems (RIBSP), Kazakhstan. The attenuated vaccinal strains Nigeria 75/1 (GenBank: KY628761.1) of PPRV and NISKhI (GenBank: AY077834.1) of SPPV were used in its production. The vaccine was tested for sterility, purity, and identity, according to OIE Terrestrial Code, 2018 (Chapter 1.1.9) (World Organization for Animal Health (OIE), Paris, France, 2018) [25]. The titer of the vaccine viruses of PPR and SPP in the 1.0 mL dose of the lyophilized combined vaccine was 10^3.0^ TCID_50_/mL.

### 2.2. Control Viruses

The virulent strain A (GenBank: AY077833.1) of SPPV, in the form of an organ–tissue lyophilized material, was obtained from the Microbial Collection Laboratory, RIBSP, Kazakhstan [26].

The PPR field virus strain Kentau-7 was obtained from the Microbial Collection Laboratory, RIBSP, Kazakhstan. This strain was isolated from the pathology material from a goat in the PPR outbreak in the territory of Kazakhstan in 2003 by performing sequential passaging (7 passages) of the supernatant of the affected organ of the fallen goat on a lamb kidney cell culture (LK) [27].

### 2.3. Animals

Fine-fleeced sheep of the Kazakh breed, aged 6–12 months, from the farms free of acute infectious diseases and seronegative to PPR and SPP were used to assess immunity duration. A total of 49 sheep were used for the research.

Prior to the experiments, the animals were labeled and held in quarantine for 1 month, with regular thermometry, clinical observation, and analysis of blood sera of specific antibodies to PPR and SPP viruses in SNT, according to OIE Terrestrial Code of 2019 (Chapter 3.7.9) and 2018 (Chapter 3.7.12), respectively [10,28]. The animals free of specific antibodies to the PPR and SPP viruses and not vaccinated against these diseases were used in the experiment.

The experiments were carried out in specially equipped ABSL-2 animal facilities. On arrival in the RIBSP animal facilities, sheep were ear-tagged and allowed to acclimatize in the facilities for two weeks prior to the onset of the experiment. Each group was housed in a separate room with no direct contact with each other. Experimental animals had free access to water and feed throughout the experiment.

This study was conducted in accordance with national and international laws and guidelines for the handling of animals. The protocol was approved by the Ethics Committee for Animal Experimentation at the RIBSP, Science Committee of the Ministry of Education and Science (SC MES), RK (Permission number: 3101/14).

### 2.4. Vaccination

The sheep were randomly divided into three groups as follows: I group (*n* = 9), II group (*n* = 20), and III group (*n* = 20). The experimental sheep were divided into groups using an online random number generator (Randomizer), while all the personnel involved in the research experiment did not have access to data about which group this or that animal belongs to.

Group I sheep (*n* = 9) were used to evaluate the safety of the developed combination vaccine by subcutaneous vaccination of 6 sheep with a dose containing 10^5.0^ TCID_50_/mL of each virus in a volume of 2.0 mL. The other 3 sheep were immunized with 2.0 mL of phosphate saline buffer (PBS).

All immunized animals were subjected to a clinical study through regular observations and daily rectal temperature records in order to detect post-vaccination reactions. The formation of inflammatory edemas in the form of infiltrates at the injection site sized up to 1.0 cm in diameter and being self-eliminated within 3–6 days was considered to be a positive post-vaccination reaction.

Sheep from the group II (*n* = 20) were used to evaluate the duration of immunity in animals post-vaccination with a combined vaccine against PPR and SPP. Sheep of this group inoculated subcutaneously with a single dose (2.0 mL) of vaccine. The titer of the vaccine viruses of PPR and SPP in the 1.0 mL dose of the lyophilized combined vaccine was 10^3.0^ TCID_50_/mL.

Sheep from group III (*n* = 20) served as controls (unvaccinated animals) for group II.

All the immunized animals were clinically monitored by regular observations and by recording daily rectal temperatures. Nasal, ocular, oral, and rectal swabs, as well as blood samples were taken from sheep within 14 days post-vaccination (dpv) and analyzed using RT-qPCR to monitor viral load. 

### 2.5. Serum Sample Collection

Blood samples were collected on 0, 7, 14, 21, 30, 90, 180, 270, and 360 dpv for serum antibody estimation. Serum was separated from all the blood samples and heat inactivated at 56 °C for 30 min and stored at −20 °C until further used.

### 2.6. SNT for PPRV and SPPV

The collected blood sera of immunized sheep were tested for PPR and SPP virus-neutralizing antibodies (VNA) in SNT in Vero cell line, according to the OIE Terrestrial Code, of 2019 (Chapter 3.7.9) and 2018 (Chapter 3.7.12), respectively [10,28]. The viral-neutralizing activity of sera was determined by the neutralization index, which was calculated taking into account the difference in logarithmic titers of the control and test sera in accordance with Reed and Muench method [29]. A neutralizing titer ˃10 is considered positive for PPRV, while an index ≥1.5 is positive for SPPV. The tests were performed at least twice and the average of the two tests was used for subsequent analysis.

### 2.7. c-ELISA Test for PPRV

At the above-mentioned time periods, the obtained blood sera were additionally analyzed in a competitive ELISA (c-ELISA) (ID Screen^®^PPR Competition (PPRC-4P), ID.vet, Montpellier, France) for the presence/absence of VNA to the PPRV, according to the manufacturer’s instructions. Then, OD at 450 nm was read. The unit of measurement was an S/N percentage. S/N percentage of ≤50% was considered positive, while 50–60% was considered doubtful. >60% S/N percentage was considered negative for the presence of antibodies against PPRV. All the results were recorded as Mean ± S.E.M.

### 2.8. RNA Extraction and Detection of PPRV Nucleic Acid in Blood and Swabs Samples

Virus genome RNA was extracted from the collected specimen with the help of the commercial kit for RNA extraction (ID Gene™ Mag Fast Extraction Kit (QIAGEN, Hilden, Germany), according to the manufacturer’s instructions.

The following primers were used to amplify the N gene of the PPRV: NP3 5′-GTC-TCG-GAA-ATC-GCC-TCA-CAG-ACT-3′ and NP4 5′-CCT-CCT-CCT-GGT-CCT-CCA-GAA-TCT-3′ [30]. The viral genome was assessed using the real-time PCR commercial kit (RT-qPCR) (ID Geneтм Peste des Petits Ruminants Duplex, IDvet genetics, Grabels, France), according to the manufacturer’s instructions in the thermocycling system Rotor-Gene Q (Qiagen, Germany) under the following program: (1) reverse transcription for 10 min at 45 °C, (2) polymerase activation for 10 min at 95 °C, and (3) denaturation/DNA elongation for 15 s at 95 °C/60 s at 60 °C.

### 2.9. DNA Extraction and Detection of SPPV Nucleic Acid in Blood and Swabs Samples

DNA from oral ad nasal swabs was extracted with the help of the ID Gene™ Mag Fast Extraction Kit (QIAGEN, Hilden, Germany), according to the manufacturer’s instructions.

The following primers were used to identify SPPV: F-primers INS1.1 5-AGA AAC GAG GTC TCG AAG CA-3 and R-primers INS1.2 5-GGA GGT TGC TGG AAA TGT GT-3, chosen from the genes of the membrane protein CD47 of the SPPV [31]. The viral genome was assessed using real-time PCR using the kit RT-qPCR (ID Gene™ Capripox Virus Triplex qPCR) (IDvet genetics, Grabels, France), according to the manufacturer’s instructions in the thermocycling system Rotor-Gene Q (Qiagen, Germany) under the following program: (1) polymerase activation for 10 min at 95 °C and (2) denaturation/DNA elongation for 15 s at 95 °C/60 s at 60 °C.

### 2.10. Challenge Studies

The challenges were performed on the 30th, 90th, 180th, 270th, and 360th dpv of sheep.

From 20 vaccinated sheep of group II, 10 sheep were randomly selected to challenge the field strain Kentau-7 of the PPRV and identified as group IIa. The remaining 10 sheep were used to challenge the virulent strain A of the SPPV and were identified as group IIb (Table 1).

Before the tests, the sheep of these subgroups were transported to separate, specially equipped rooms (ABSL 2), and their rectal temperature was recorded.

Of the 20 sheep of group III, 10 sheep were used to challenge the virulent strain A of the SPPV and were identified as group IIIa, and the remaining 10 sheep were used to challenge the field strain Kentau-7 of the PPRV and were identified as group IIIb (Table 1).

Before the tests, which were carried out on the 30th, 90th, 180th, 270th, and 360th days, 2 sheep from each control subgroup (IIIa and IIIb) were transported to separate, specially equipped rooms (ABSL 2), and their rectal temperature was recorded.

Vaccinated (IIa) and control (IIIa) animals selected for the challenges for the PPRV were infected with the field strain Kentau-7 of the PPRV subcutaneously in the subscapular region at a dose 1 × 10^5.0^ TCID_50_/mL (Table 1).

Vaccinated (IIb) and control (IIIb) animals selected for challenge study for the SPPV were infected intradermal in the area under the tail fold with a virulent strain A of the SPPV at a dose of 1 × 10^4.5^ TCID_50_/mL (Table 1).

Control of the experimental animals (vaccinated and intact sheep) were carried out within 14 days post-challenge (dpс), with daily measurement and registration of rectal temperature and for the manifestations of clinical signs of PPRV and SPPV, which were evaluated using the point systems, described earlier [32,33]. Nasal, ocular, oral and rectal swabs, as well as blood samples were taken from sheep within 14 dpс and analyzed using RT-qPCR to monitor viral load.

After completing the control trials, the recovering control sheep were treated with antibiotics to avoid secondary infection and sheep that reached clearly defined humane endpoints of a moderate severity were euthanized humanely.

### 2.11. Statistical Methods

Statistical analysis was carried out with the use of GraphPad Prism version 8.0.1. The results of the serological test, rectal temperatures post sheep vaccination with both vaccinal strains, as well as the difference between groups after the challenge with control viruses were analyzed with the help of bilateral ANOVA tests. A value *p* ≤ 0.05 was considered statistically significant. The difference in efficacy between the groups was compared using the one-lateral Fisher’s exact test for two proportions at the alpha level of ˂0.05.

## 3. Results

### 3.1. Safety of the Developed Vaccine

**Figure 1:**
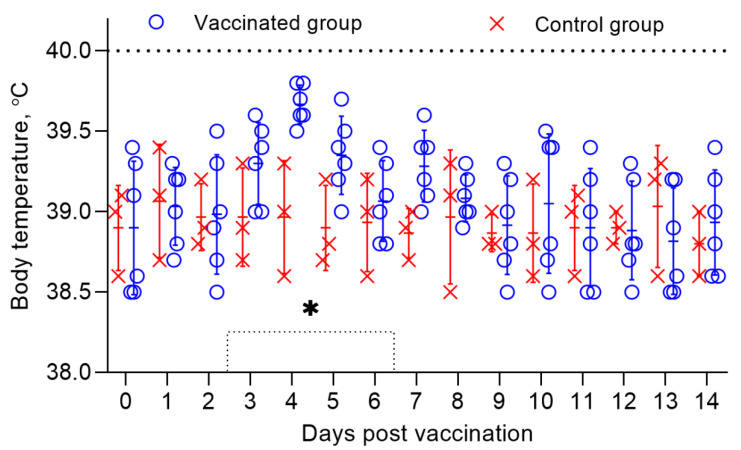
Body temperature of vaccinated sheep when studying the safety of a combined vaccine against PPR and SPP. The dotted line in the graph shows the upper limit of the normal body temperature. Rectal temperatures of 6 vaccinated animals and 3 control animals are shown in the graph. (*) Local reactions that appeared post vaccination in 2 vaccinated sheep existed for 4 days.

The general post-vaccination condition of the vaccinated sheep within 3 weeks was within normal limits. Only two sheep had local reactions in the form of irregularly rounded swellings, 0.5 cm in diameter, pinkish in color, and protruding 3 mm above the surface of the surrounding skin. The infiltrates gradually disappeared within 4 days without an increase in body temperature. The body temperature of both vaccinated and control sheep remained within the normal range (38.5–39.8 °C) (Figure 1) for 14 days.

### 3.2. SNT Results for PPRV

**Figure 2:**
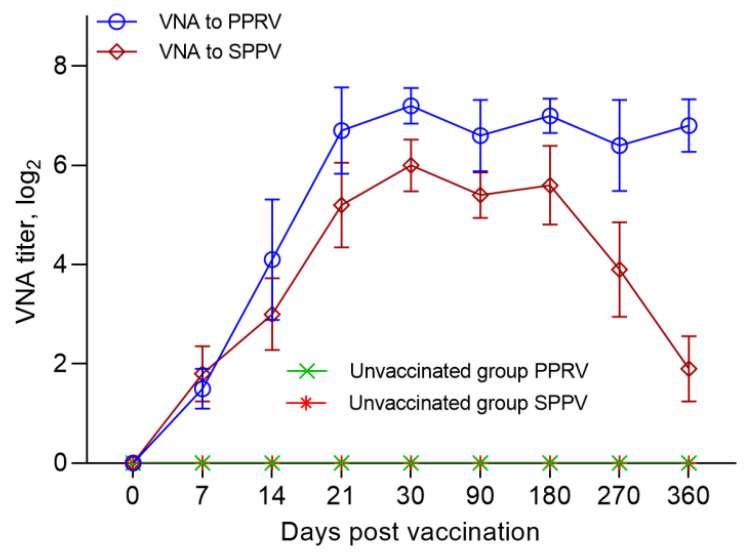
Neutralizing antibody responses in sheep following vaccination with a combination vaccine. Blood sera of vaccinated sheep were taken and tested in a SNT at 0, 7, 14, 21, 30, 90, 180, 270, and 360 dpv. Neutralizing titers were determined against Nigeria 75/1 vaccine strain and NISKhI vaccine strain using specific sera for the PPRV and for the SPPV. All vaccinated sheep (100%) were protected against PPRV and SPPV at 21 dpv and 14 dpv, respectively.

On the day of vaccination (day 0), all the animals were seronegative for PPRV antibodies. After 7 days, NAs with an average titer of 1.5 log2 was detected in the serum samples of the immunized sheep, which increased to 4.1 log2 on the 14th dpv. On day 21, the NAs titer reached 6.7 log2. The peak of the average NAs titer (7.2 log2) in the blood serum samples of immunized sheep was recorded on the 30th dpv. The indicated NAs titer was maintained up to 360 dpv, with slight fluctuations (*p* ˃ 0.05) (Figure 2). Antibodies to PPRV were absent in the blood sera of the control sheep (Figure 2).

### 3.3. SNT Results for SPPV

On the day of vaccination (day 0), all the animals were seronegative for SPPV antibodies. A week after vaccination, the sheep were found to have NAs with an average titer of 1.8 log2. From day 7 to day 21, there was a steady increase in the average NA titers in the blood sera of vaccinated sheep. Thus, on the 14th and 21st dpv, NAs titer in the blood serum of immunized sheep reached 3.0–5.2 log2, respectively. The highest NAs titer (6.0 log2) in the blood sera of immunized sheep was detected at 30 dpv. The developed antibodies were preserved for up to 6 months post vaccination, with insignificant fluctuations (*p* ˃ 0.05). Further, a steady decrease in the titer of the SPPV was observed, and by the end of the experiment (after 360 days), it was only 1.9 log2 (*p* ˂ 0.001) (Figure 2). Antibodies of SPPV were absent in the blood sera of control sheep (Figure 2).

### 3.4. с-. ELISA Test Result for PPR

**Figure 3:**
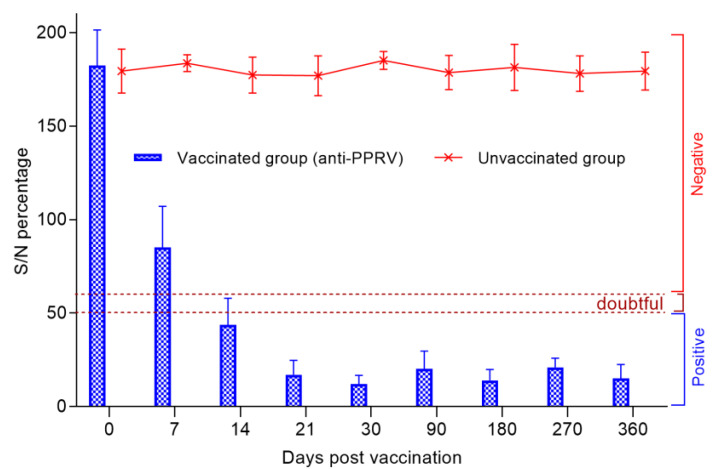
Evolution of the average percentage of inhibition (with standard deviation) in each group of vaccinated and control sheep during the experiment after a single vaccination. In the present study, the unit of measurement was S/N percentage, and it was assumed that a lesser S/N % meant higher antibody levels. S/N percentage of ≤ 50% was considered positive, while that of 50 ˂ S/N ≤ 60% was considered doubtful. >60% S/N percentage was considered negative for the presence of antibodies against PPRV. All the results were recorded as Mean ± S.E.M.

Before vaccination, sheep serum samples were tested for the presence of antibodies, which were found to be negative, as all the samples had an S/N ratio >150%. After being vaccinated for only 7 days, the percentage of S/N dropped sharply to 85%, with 45% of the animals being protected, the remaining 25% of the animals had a titer of produced antibodies above the permissible limit of the S/N value (˃60%), while in 30% of the animals, the c-ELISA results were questionable (50–60%). However, at the 14th dpv, 95% of the vaccinated sheep were protected from PPRV, judging by the antibody titers, since the S/N values were 50%. The average S/N value was 17% on the 21st dpv, and all animals were 100% protected from PPRV. The lowest S/N % value (12%) was recorded on the 30th dpv. The antibodies produced by day 30 post-vaccination persisted until the end of the experiment (360 days), with slight fluctuations (*p* ˃ 0.05) (Figure 3).

### 3.5. Detection of Viral Nucleic Acids in Blood and Swabs

In all the blood samples and swabs taken from vaccinated sheep (group II) post-vaccination, no viral nucleic acids specific to the PPRV and SPPV were detected, indicating that the combined vaccine was safe to use and provided sterile immunity against both viruses. By contrast, in all the types of samples taken from unvaccinated animals, PPRV and SPPV specific viral nucleic acids were detected.

**Figure 4:**
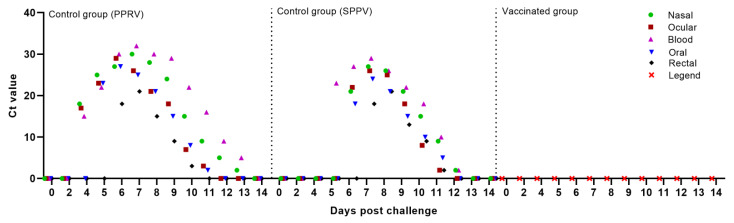
PPRV-specific RNA and SPPV-specific DNA were measured by real-time reverse transcription PCR (RT-qPCR), and the amount of viral RNA and DNA is expressed as a Ct, a value that increases as the amount of viral RNA increases.

Starting from 2–3 days post-infection with the field strain Kentau-7 from the nasal swabs of all unvaccinated sheep, from 4–5 days from the blood and ocular swabs in 6 out of 10 tested sheep, from 5–6 days from oral swabs in 8 out of 10 tested sheep, as well as from 6–7 days from rectal swabs in 4 out of 10 infected sheep, viral nucleic acid specific for the PPRV was detected (Figure 4).

Capripoxvirus genomes were first detected in the sheep’s blood samples on 5–6 days post-challenge (dpc) with virulent strain A, whereas in most infected animals, viral DNA was first detected in nasal, ocular, and oral swabs at 6–7 days. Note that the infectious virus was detected in the nasal and ocular secretions of all unvaccinated sheep, while in oral swabs, viral DNA was isolated in 7 out of 10 infected sheep. In rectal swab samples, viral DNA was detected from day 7 post challenge in 7 out of 10 sick sheep (Figure 4).

### 3.6. Clinical Protection Post Challenge with the PPR Field Virus Strain Kentau-7

**Figure 5:**
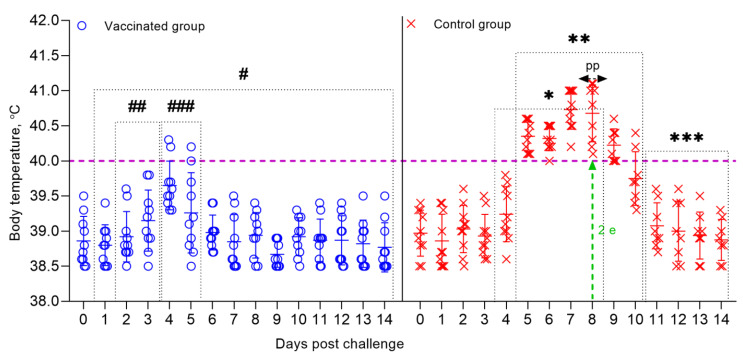
Dynamics of changes in the body temperature of sheep that are vaccinated, and control groups post-challenge with the field strain Kentau-7 of the PPRV. Rectal temperatures of ten animals in each group are shown in the graph. The dotted line in the graph shows the upper limit of the normal body temperature. (#) Within 14 days, in the vaccinated animals, no clinical symptoms characteristic of PPRV were observed, with the exception of local reactions in three sheep. (##) Local reactions that appeared post-challenge in vaccinated sheep existed for 2 days. (###) Temperature reactions that appeared post-challenge in vaccinated sheep existed for 2 days. (*) Starting on the 3rd dpc, unvaccinated sheep developed local reactions in the form of swellings. (**) From 5 to 11 days, the control animals developed clinical symptoms, such as pyrexia in the range of 40.0–40.6 °C, watery and mucopurulent discharge from the eyes and nose, hyperemia in the gums, and some animals had diarrhea, decreased appetite, and depression. (pp) The peak of pyrexia in unvaccinated animals was noted on the 8th dpc. (2 е) On day 8, 2 sheep in the control group were humanely euthanized post challenge due to the deterioration of their general condition. (***) During the specified time intervals (from 10 to 14 days), 8 out of 10 infected animals remained alive and began to recover.

**Figure 6:**
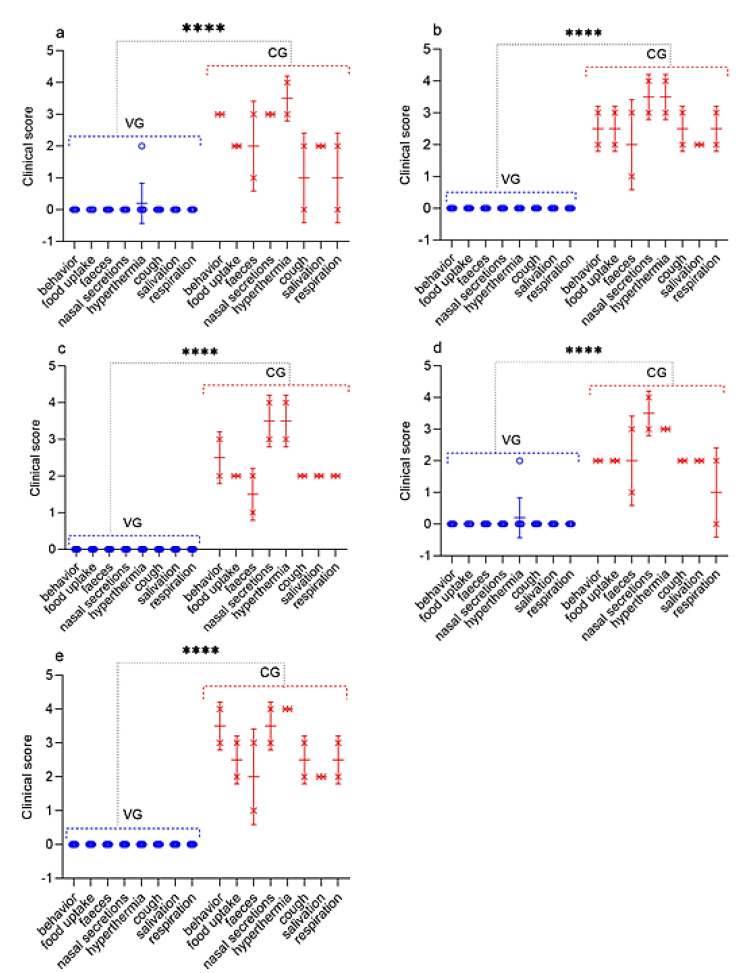
Assessment of the clinical signs in vaccinated and control sheep post challenge with the field strain Kentau-7 of the PPRV. (**a**) Challenge 30 dpv. (**b**) Challenge 90 dpv. (**c**) Challenge 180 dpv. (**d**) Challenge 270 dpv. (**e**) Challenge 360 dpv. VG vaccinated group infected with virulent SPPV (*n* = 10). CG control group infected with virulent SPPV (*n* = 2). Data are means ± standard errors; **** *p* < 0.0001.

The vaccinated sheep did not show clinical symptoms of PPR, and their rectal temperature did not exceed the standard limits (38.5–39.8 °С), except for two sheep that underwent challenged on 30 dpv and 270 dpv (Figure 5); at this stage, their clinical score reached up to 1 (Figure 6). At the same time, in one vaccinated sheep that passed the control test 30 dpv, showed an increase in body temperature of 40.2 °C for 2 days post-challenge, which then returned to normal (Figure 5). In one vaccinated sheep that passed the control test, 270 dpv, the body temperature reached 40.3 °C at 4 dpc, which returned to normal at 6 dpc (Figure 5). In two sheep that passed the control test at 90 dpv, local reactions were noted in the form of wrong-rounded shape swellings, measuring 0.1 and 0.2 cm in diameter, pink in color, and protruding 1 mm above the surface of the surrounding skin, which were independently stopped within 2 dpc. However, the samples taken from the local reactions of vaccinated sheep, as a result of the PCR analysis, were clean.

### 3.7. Necropsy of Dead and Euthanized Sheep Post Challenge with the Field Strain Kentau-7 of the PPRV

All control animals began to get sick on the 5th dpc, with the development of pyrexia in the range of 40.0 to 40.6 °C. In most infected animals, pyrexia lasted up to 9 days. The peak of pyrexia to 41.1 °C (equal to 4 clinical points) in three infected animals was observed on 8 dpc, after which the temperatures of sick animals began to decrease (Figure 5; Figure 6).

**Figure 7:**
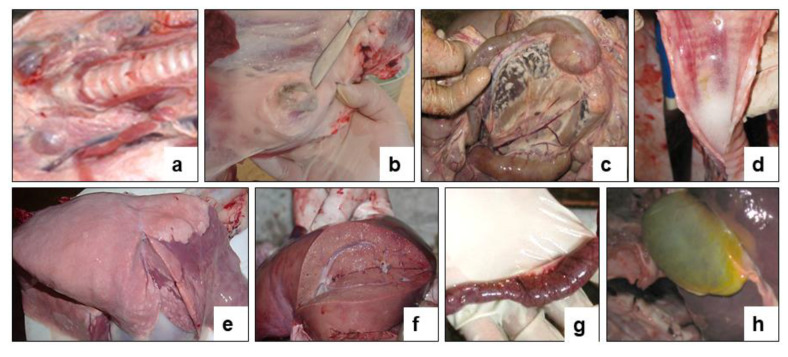
Internal examination of forcibly euthanized sheep post challenge with the field strain Kentau-7 of the PPRV. (**a**) Enlargement of the pharyngeal lymph nodes. (**b**) Enlargement of the pre-scapular lymph node. (**c**) Enlarged mesenteric lymph node on the incision. (**d**) Congestive hyperemia and isolated spot hemorrhages in the mucous membranes of the larynx and trachea. (**e**) Spotty hemorrhages under the serous membrane of the lungs. (**f**) Hemorrhages under the serous membrane of the liver. (**g**) The mucous membrane of the small intestine is swollen, reddened and covered with mucus (signs of acute catarrhal enteritis). (**h**) The gallbladder is full.

All infected sheep showed clinical signs specific to PPRV in the form of watery discharge from the eyes and nose and local reactions in the form of swellings, measuring 1.0–2.0 cm in diameter. In two control sheep, watery discharge from the eyes and nose gradually became purulent on 7–8 dpc. In addition, within 4 days of the onset of the fever, the gums of all the animals became hyperemic. In general, the animals were lethargic and depressed. Additionally, some animals had diarrhea, cough, but they were not dominant. At this stage, each sheep had a total clinical score ranging from 10 to 23. Two sheep that passed the control test at 90 dpv and 360 dpv had respiratory disorders in the form of pneumonia and had periodic coughing in connection with this; their total clinical scores reached 25–26. However, despite the manifestation of pronounced clinical signs specific to PPRV, of the 10 control animals, 8 animals recovered. After conducting control tests (14 days), the recovered animals were isolated and treated with antibiotics in order to avoid the appearance of secondary infections. Two sheep from the control group (selected on the 90th and 360th day) were humanely euthanized on the 8th dpc due to the deterioration of their general condition; at this stage, they achieved clinical indicators of 25 and 26, respectively (Figure 6). Forcefully euthanized sheep were subjected to a necropsy (Figure 7).

The necropsy of euthanized control animals showed hyperemia and enlargement of the pharyngeal (Figure 7a), pre-scapular (Figure 7b) and mesenteric (Figure 7c) limph nodes. Hemorrhages were found in the tracheal mucosa (Figure 7d), in the lungs (Figure 7e), and in the liver (Figure 7f). The mucous membrane of the small intestine was thickened in places, covered with gray-brown mucus, and there were hemorrhages all over the surface of the intestinal mucosa (Figure 7g). The gallbladder was filled (Figure 7h). Samples taken from the affected organs (lungs, liver, and intestines) were positive for PPRV in RT-qPCR studies.

### 3.8. Clinical Protection Post Challenge with the Virulent Strain A of the SPPV

**Figure 8:**
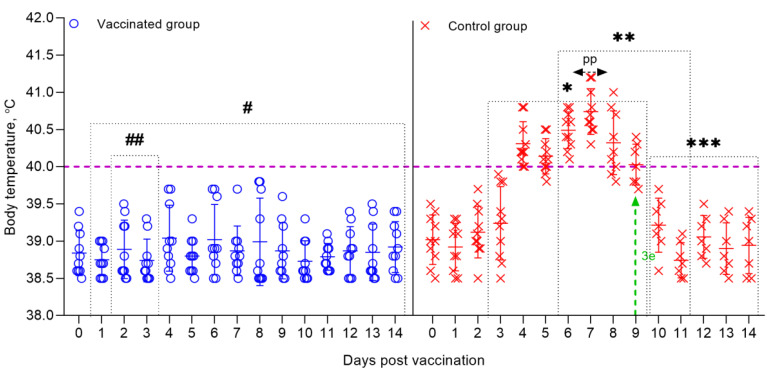
Dynamics of changes in the body temperature of sheep vaccinated and control groups post-challenge with the virulent strain A of the SPPV. Rectal temperatures of ten animals in each group are shown in the graph. The dotted line in graph shows the upper limit of the normal body temperature. (#) Within 14 days, in the vaccinated animals, no clinical symptoms characteristic of SPPV were observed, with the exception of local reactions in three sheep. (##) Local reactions that appeared post challenge in vaccinated sheep existed for 2–3 days. (*) Starting on the 2nd dpc, unvaccinated sheep developed local reactions in the form of edema of a dense consistency. (**) From 5 to 12 days, the control animals developed clinical symptoms, such as watery and mucopurulent discharge from the eyes and nose, papules, hyperemia in the oral cavity, decreased appetite, and depression. (3 e) On day 9, 3 sheep from the control group were humanely euthanized post-challenge due to the deterioration of their general condition. (pp) The peak of pyrexia in unvaccinated animals was noted on the 7th dpc. (***) During the specified time intervals (from 10 to 14 days), 7 out of 10 infected animals remained alive and began to recover.

In immunized sheep post challenge (after 30, 90, 180, 270, and 360 days), no clinical symptoms of smallpox infection were observed, the body temperature of the sheep was within the physiological norm (38.5–39.7 °C) (Figure 8), except for local reactions in three sheep at the site of the introduction of the virulent virus SPP. At the same time, in one vaccinated sheep that passed the control test 30 dpv, the local reaction, equal to one clinical score, was in the form of a speck, pink in color and in 1.0 cm in diameter, which independently disappeared at 3–4 dpс. In two vaccinated sheep that underwent control testing 270 dpv, local reactions were in the form of wrong-rounded shape swellings, measuring 0.5 cm and 1.0 cm in diameter, which were resolved on the 3nd and 4rd dpс, respectively.

**Figure 9:**
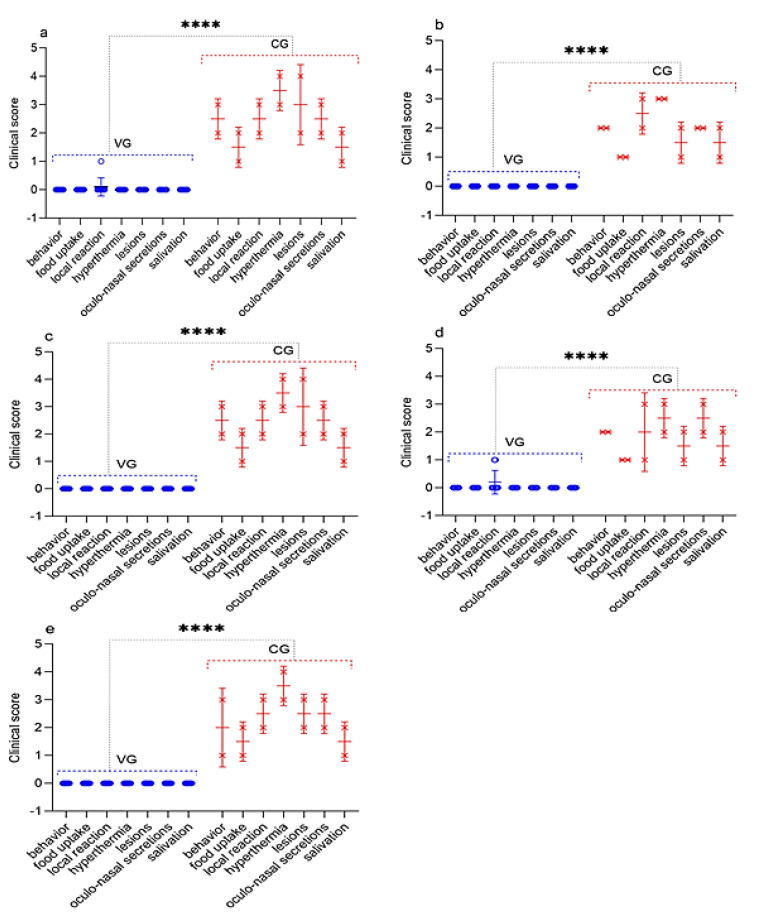
Assessment of the clinical signs in vaccinated and control sheep after infection with virulent SPPV. (**a**) Challenge 30 dpv. (**b**) Challenge 90 dpv. (**c**) Challenge 180 dpv. (**d**) Challenge 270 dpv. (**e**) Challenge 360 dpv. VG vaccinated group infected with virulent SPPV (*n* = 10). CG control group infected with virulent SPPV (*n* = 2). Data are means ± standard errors; **** *p* < 0.0001.

In animals infected with the virulent SPPV (strain A), primary clinical lesions began to develop on 2–3 days dpc, with the development of a local reaction in the form of an edema of a dense consistency up to 3 × 4 – 4 × 5 cm in size. At this stage, the clinical score of each sheep increased by 1–3 points (Figure 9). On day 5, the body temperature of infected sheep reached 40.8 °C. The peak of pyrexia of 41.2 °C (equal to 4 clinical points) was observed on the 7th dpc in three sheep (Figure 9). The body temperature in seven sick animals began to normalize on the 11–12 dpc (Figure 8).

**Figure 10:**
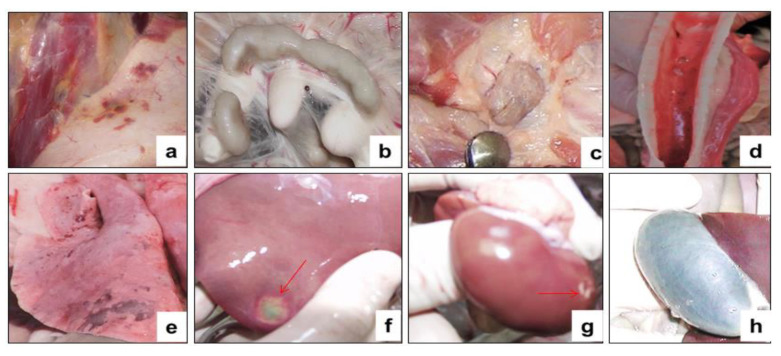
Internal examination of forcibly euthanized sheep post-challenge with the virulent strain A of the SPPV. (**a**) Spotty hemorrhages in the subcutaneous tissue and musculature. (**b**) Enlarged mesenteric lymph nodes. (**c**) Enlargement of the popliteal lymph nodes. (**d**) Subcapsular lesions in the tracheal mucosa. (**e**) Spotty hemorrhages, and lesions in the lungs. (**f**) Serous lesion in the liver. (**g**) Subcapsular lesion in the kidney. (**h**) The gallbladder is full.

On the 5th day, simultaneously with the fever, all experimental animals had watery and mucopurulent discharge from the eyes and nose, as well as skin lesions (papules) with a diameter of 1.0–2.0 cm on the inner surface of the skin of the forelimbs and hind limbs, as well as on the skin of the udder. Most of the sick animals (70%) had a loss of appetite, which indicated the presence of hyperemia in oral cavity. At this stage, each sheep had a total clinical score in the range from 10 to 18. Additionally, on day 9, three sheep from the control group (selected on day 30, 180, and 360) were humanely euthanized post-challenge due to the deterioration of their general condition, presenting, respectively, a clinical score of 21, 21, and 20 (Figure 9). In addition to the above clinical signs, shortness of breath, cough, and moderate to severe depression were observed in the euthanized sheep. The corpses of the euthanized sheep were subjected to a necropsy (Figure 10). After conducting control tests (14 days), the recovered animals were isolated and treated with antibiotics in order to avoid the appearance of secondary infections.

### 3.9. Necropsy of Dead and Euthanized Sheep Post-Challenge with the Virulent Strain A of the SPPV

During the autopsy of the euthanized sheep, pinpoint hemorrhages were found in the subcutaneous tissue and musculature (Figure 10a). The lymph nodes throughout the body were mostly enlarged and swollen, especially the popliteal lymph nodes (Figure 10b,c). Small, well-defined, pale subcapsular lesions were found in the tracheal mucosa of the autopsy animals (Figure 10d). The lungs were swollen, especially in the dead sheep, and had spotty hemorrhages under the serous membrane of the lungs and lesions with foci of gray nodules (Figure 10e). Lesions were found in the liver and kidneys (Figure 10f,g). The gallbladder was filled (Figure 10h). The results of the RT-PCR showed that samples taken from the affected organs (lungs, liver, and kidneys) of euthanized animals were positive for SPPV.

## 4. Discussion

Today, sheep breeding is considered to be one of the most important branches of the agro-industrial complex of Kazakhstan, which in some cases is the only source of the most important types of products: wool, lamb meat, fur, and fur sheepskins. The number of sheep in Kazakhstan is increasing every year, and according to the Bureau of National Statistics of the Agency for Strategic Planning and Reforms of the Republic of Kazakhstan, at the beginning of this year, the number of sheep was 20.05 million heads. However, as has been established, a large concentration of animals in limited areas coming from different epizootic regions, a wide exchange of animals within the country and the import of their highly productive breeding breeds from abroad, difficulties in organizing continuous production, full-fledged feeding and ensuring an optimal microclimate create favorable conditions for the occurrence of mass infectious diseases. These dangerous infectious diseases of animals include PPR and SPP.

PPR and SPP are highly infectious diseases of small ruminants, which, in the event of an outbreak, cause significant economic damage to agriculture. An outbreak of these infections has been registered in countries bordering Kazakhstan, including Russia and China. The epizootic situation that has developed to date for these infections indicates the need to develop a highly effective means of specific prevention against both PPR and SPP. In this regard, we have developed a combined vaccine for the simultaneous prevention of PPR and SPP.

The objective of this work was to assess the duration of immunity in sheep that were vaccinated with a combined vaccine against PPR and SPP developed in the Republic of Kazakhstan. 

Previously, the mass vaccination of animals using this type of vaccine against PPR and SPP was reported [34], where the authors revealed a difference in sensitivity between sheep breeds to the PPRV and SPPV, which affected the level of seropositivity in animals to these viruses.

In our study, when testing SN, the seropositivity of the fine-fleeced Kazakh breed for the SPPV virus reached 95% at 7 dpv and 100% at 14 dpv, while at 14 dpv, the seropositivity to PPRV reached 80% and 100% protection of animals from PPRV, and this was noted at 21 dpv (Figure 2). The serological response to PPRV obtained using c-ELISA reached 95% at 14 dpv and 100% at 21 dpv (Figure 3).

Protective titers of NA to SPPV (1.8 log2) developed in vaccinated sheep, one week earlier, as compared to the titers of NA to PPRV (4.1 log2), which were formed on the 14th day of dpv (Figure 2). Similar results were obtained in the study by Fakri et al. [21,22] where the authors observed an early humoral response (after 7 days) to SPPV post-vaccination of animals with a combined vaccine. However, only 90% of the vaccinated animals were protected from SPPV 21 dvp with this vaccine.

Immunity against PPRV formed in animals vaccinated with the combined vaccine persisted for 12 months without a significant decrease in antibody titers (6.8 log2) (*p* ˃ 0.05), while SPPV titers persisted for up to 6 months (5.6 log2) (*p* ˃ 0.05), and then gradually decreased until the end of the experiment (1.9 log2) (Figure 2). However, the results of the study on the developed vaccine on a control model demonstrated the ability of this vaccine to induce 100% clinical protection against the field PPRV and against the virulent SPPV until the end of the experiment (12 months) (Figure 2 and Figure 3). During control tests, two vaccinated animals, that passed the control test at 30 dpv and 270 dpv, showed a slight increase in body temperature post-challenge with the field strain Kentau-7 of the PPRV, whereas the unvaccinated animals developed typical clinical signs of the disease (Figure 5). The vaccinated animals had an average clinical score of 0.2 points post-challenge with the field strain Kentau-7 of the PPRV, whereas the unvaccinated animals had an average score of 19.3 points, indicating the high protective activity of the test vaccine against the field strain of PPRV.

As is known in many studies, when evaluating the protection provided by PPR vaccines, vaccinated and control animals are infected intranasally (i/n), as it is believed that PPRV, like other morbilliviruses, initially infects the epithelial cells of the respiratory tract. However, Pope, R.A. et. al. (2013) suggest that the virus is absorbed by the immune cells of the mucous membrane of the respiratory tract, which then transfer the virus to the lymphoid tissues, where the primary replication of the virus occurs and from where the virus enters the bloodstream [35].

Note that the i/n of infection of animals has additional difficulties associated with the fact that animals shake their heads/sneeze after the introduction of a virulent virus, which can lead to an insufficient dosage of the virus, or to an overdose of the virus, as when sneezing infected animals release the virus into the environment and animals (especially with weaker immunity) can receive an additional dose of the virus. In this regard, we choose the subcutaneous route of infection for PPR, instead of the intranasal.

In this regard, in order to challenge the strain Kentau-7 PPRV, we chose the subcutaneous route of infection, instead of the intranasal. In our studies, pronounced clinical signs of PPR were observed in animals subcutaneously infected with the field strain Kentau-7 PPRV (Figure 6 and Figure 7).

Furthermore, three vaccinated sheep post-challenge with the virulent strain A of the SPPV had local reactions, which disappeared independently within 3–4 days, whereas the unvaccinated animals developed typical clinical signs of the SPP (Figure 9 and Figure 10). For the vaccinated and control groups of animals post-challenge with the virulent strain A of the SPPV, the average clinical score for the entire study period was calculated, the value of which was 0.2 points for the vaccinated group, and 15.2 points for the control group.

Blood and swab samples were collected from the animals following vaccination and challenge, and RT-qPCR was carried out to detect viral nucleic acids in blood and various body excretions. None of the vaccinated animals showed the presence of PPRV and SPPV in blood samples and nasal, ocular, oral, and rectal swabs collected at regular intervals after vaccination and challenge. However, after challenge, the PPRV and SPPV antigen was detected in the blood and in all types of swabs taken from intact animals (Figure 4). Similar results were obtained by S. S. Chaudhary et al. (2009), as well as by M. Hosamni et al. (2006), when using a divalent vaccine against PPRV and SPPV/ GPV [17,36].

Thus, we conclude that a single immunization of sheep with the recommended dose (10^3.0^ TCID_50_/mL) of the developed combined vaccine against the PPR and SPP induces immunity against the SPPV on day 7 and against the PPRV on day 14 post-vaccination. The combined vaccine provides reliable protection against two infections simultaneously for 12 months (observation period). At the same time, in sheep vaccinated with this vaccine, immunity against PPRV persists for more than 12 months. This is due to the choice of the vaccine strain Nigeria 75/1, which, as the main component of a live monovalent vaccine, causes strong immunity in vaccinated animals, lasting at least 3 years. Note that the Niskhi vaccine strain is able to cause earlier humoral immunity in vaccinated animals compared to other vaccine strains of the SPPV, which are used to make a combined vaccine and provides 100% protection against SPP for up to 12 months.

The obtained results once again proved the effectiveness and safety of the combined vaccines against PPR and SPP while being used, thus disproving the incompatibility of the vaccine strains of PPRV and SPPV. As has been established, Rajak et al. (2005), in their earlier studies, have shown that the vaccinal PPRV does not influence the immunogenicity of other unrelated antigens, which is confirmed by our results and the results obtained by Fakri et al. (2020c) that demonstrated prolonged compatibility of PPRV and SPPV [34,37].

Future studies should examine the effectiveness of the combined vaccine in the field for large vaccination campaigns.

## 5. Conclusions

Based on the analysis of the research data we obtained, we can conclude that the developed combined vaccine against PPR and SPP provides reliable protection against these infections in immunized sheep for 12 months. At the same time, vaccinated animals have immunity against PPR for more than 12 months. Large-scale field trials using the developed combined vaccine, as well as in increasing the immunogenicity of the NISKhI strain of the SPP virus, need further study. The developed combined vaccine can be an alternative to the monovalent vaccines against PPR and SPP used in Kazakhstan and in Asian countries, which, when used, protect vaccinated animals from PPR and SPP for up to 12 months.

## Figures and Tables

**Table 1:** Design of the challenges on animals. s/c: subcutaneously; i/d: intradermal.

Challenge Virus	Group	Number of Animals	Dose and Method of Infection
Field strain Kentau-7 of the PPRV	Gr- IIa	10	1 × 10^5.0^ TCID_50_/mL, s/c
	Gr- IIIa	10	
Virulent strain A of the SPPV	Gr- IIb	10	1 × 10^4.5^ TCID_50_/mL, i/d
	Gr- IIIb	10	

## Data Availability

Not applicable.
